# Microbiome and metabolic disease: revisiting the bacterial phylum Bacteroidetes

**DOI:** 10.1007/s00109-016-1492-2

**Published:** 2016-11-29

**Authors:** Elizabeth L. Johnson, Stacey L. Heaver, William A. Walters, Ruth E. Ley

**Affiliations:** 1 Department of Molecular Biology and Genetics, Cornell University, Ithaca, NY 14853 USA; 2 Department of Microbiome Science, Max Planck Institute for Developmental Biology, 72076 Tübingen, Germany

**Keywords:** Obesity, Gut microbiome, Bacteroidetes, Type 2 diabetes

## Abstract

Bacterial species composition in the gut has emerged as an important factor in obesity and its related metabolic diseases such as type 2 diabetes. Out of thousands of bacterial species-level phylotypes inhabiting the human gut, the majority belong to two dominant phyla, the Bacteroidetes and Firmicutes. Members of the Bacteroidetes in particular have been associated with human metabolic diseases. However, their associations with disease are not always consistent between studies. Delving deeper into the diversity within the Bacteroidetes reveals a vast diversity in genomes and capacities, which partly explain how not all members respond equally to similar environmental conditions in their hosts. Here, we discuss the Bacteroidetes phylum, associations of its members with metabolic phenotypes, and efforts to characterize functionally their interactions with their hosts. Harnessing the Bacteroidetes to promote metabolic health will require a nuanced understanding of how specific strains interact with their microbial neighbors and their hosts under various conditions.

## Introduction

The human gut microbiome is composed of roughly 1.5 Kg of cells, most of which are bacterial, with a minority belonging to Archaea (e.g., methanogens) and Eukaryotes (e.g., yeasts) [1]. In Western populations, the phyla Bacteroidetes and Firmicutes are generally dominant in the gut, with other phyla comprising 10% or less of the microbiome [1]. The Bacteroidetes range in relative abundance across individuals, but generally make up half or more of the gut microbiome [1, 2]**.** Members of the Bacteroidetes mostly inhabit the distal gut, where they participate in provisioning the host with energy harvested from the diet through the fermentation of otherwise indigestible polysaccharides. This activity produces short-chain fatty acids (SCFAs) that can supply up to 10% of daily calories when the diet is rich in fiber [3, 4]. Members of the Bacteroidetes and Firmicutes occupy different functional niches in the gut ecosystem. As a result, differences between individuals in their relative proportion can lead to large differences in function, with relevance for host health.

The idea that the composition of the gut microbiome would influence host metabolism was first investigated directly by Jeffrey Gordon at Washington University School of Medicine on the basis of three key observations. First, compared to lean littermates, genetically obese (leptin deficient) mice harbored half as many Bacteroidetes in their ceca [5]. Second, metagenomic analysis revealed that the microbiomes of these lean and obese mice encoded a different proportion of metabolic pathways. When transferred to previously germfree mice, the obese-mouse microbiomes promoted greater fat gain in recipients compared to microbiomes of lean donor mice [6]. Metabolomic profiling supported the hypothesis that the obese-associated microbiome liberates more energy from the diet compared to the lean-associated microbiome, thereby contributing to the obese state. Third, a link to human health came from a study of fecal diversity in relation to weight loss in obese subjects [7]. Twelve human obese subjects were enrolled in a year-long weight loss study that included a reduction in intake of dietary carbohydrate or fat. Over the course of the year, on average, the subjects lost weight and relative levels of Bacteroidetes increased in their feces, regardless of their specific diet. Together with the mouse studies, these results suggested that the microbial ecology of the gut was dynamically linked to the obese state and could contribute to it by modulating energy harvest from the diet.

Since these initial observations, the understanding of what factors drive levels of Bacteroidetes in the gut has evolved, along with insights into their diversity, metabolism, and behavior under different conditions. A number of research groups have addressed the question of whether the ratio of Firmicutes to Bacteroidetes is a marker for obesity, either directly or as part of larger studies. Meta-analyses of these collective datasets have revealed this overall pattern: within a study, obese and lean microbiomes can be differentiated using 16S rRNA gene sequence data [8, 9]. However, the specific aspect of the data (e.g., Firmicutes to Bacteroidetes ratio, or species richness) that differentiates lean and obese microbiomes is not always the same between studies. A lower proportion of the phylum Bacteroidetes in the obese gut microbiome may or may not emerge from comparisons of lean and obese individuals. This has been discussed elsewhere [8, 9] and may relate to methodological differences between studies and/or relate to the fact that obesity, as defined by a high BMI, is a poor proxy for high adiposity, and that obese subjects vary tremendously in their health states. However, regardless of how phylum-level patterns track with obesity, specific members can nevertheless have profound influences on host metabolism. Here, we revisit the phylum Bacteroidetes and the connections that its various members have with obesity, diet, and associated metabolic diseases.

## The phylum Bacteroidetes

**Fig. 1.**
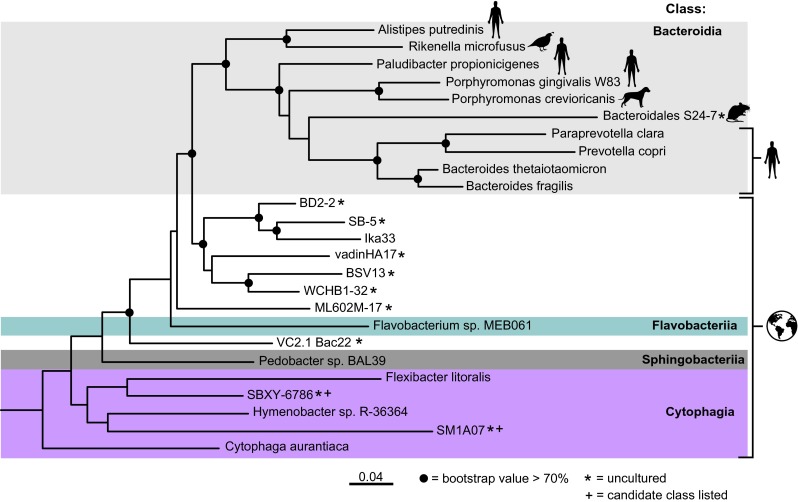
Phylogeny of the Bacteroidetes phylum shows human-associated genera are derived from environmental clades. The 16S rRNA gene sequences used to build this phylogeny were chosen to include representatives of each class within the Bacteroidetes phylum. To add focus on common human-associated Bacteroidetes, additional sequences were included for *Alistipes*, *Prevotella*, *Bacteroides*, S24–7, *Rikenella*, *Porphyromonas*, and *Paraprevotella*. *Dictyoglomus thermophilum* was used as an outgroup. The tree was built as follows: aligned 16S rRNA sequences (>1300 nt), with high entropy and gapped positions filtered, were used as input for a maximum likelihood phylogenetic estimation in RAxML (assuming a GTR + Υ model of evolution). Nodes on the tree represent >70% bootstrap support (100 replicates). Symbols (human, earth, etc) show the provenance of the sequences. *Scale bar units* are substitutions/site

What is the phylum Bacteroidetes? Understanding of this important group comes principally from two sources: characterization of cultured isolates and culture-independent sequence-based analysis of samples. Sequence-based phylogeny has superseded phenotypic descriptions in the classification of microbes, and the most widely used phylogeny is that of the small subunit ribosomal RNA gene (16S rRNA for Bacteria and Archaea, 18S for Eukaryotes [10]). By definition, bacteria belonging to the same phylum share a common ancestor, and this is represented in the bacterial phylogeny as a single basal node. 16S rRNA gene sequences that comprise a phylum generally differ in pair-wise sequence identity with those of other phyla by 30% or more, but a shared ancestry is the primary determinant for belonging to a phylum. Figure 1 shows a 16S rRNA gene phylogeny for the phylum Bacteroidetes built from a very small sampling of the tens of thousands of sequences belonging to the phylum that can be obtained from references databases such as SILVA and Greengenes [11, 12]. Here, we focus on three clades of the tree that correspond to the three predominant Bacteroidetes genera of the human GI tract: *Bacteroides*, *Prevotella*, and *Porphyromonas*, although others such as *Alistipes* and *Parabacteroides* are no doubt very important in their own right.

The structure of the Bacteroidetes phylogeny suggests that the mammal-associated taxa in the phylum Bacteroidetes are derived from environmental taxa [13]. Figure 1 shows phylogeny built from a subset of taxa belonging to this phylum. For the sake of clarity, we selected one sequence from each class within the phylum, except for the taxa associated with human hosts. This small representation is enough to capture the large-scale (class-level) topology of the phylum. What is apparent from the large-scale topology of the tree is that the mammal-associated taxa are the most derived—they are the latest to branch off and have the longest branch lengths. This is consistent with the notion that mammalian gut Bacteroidetes are derived from ancestors that once were free living in the environment and likely co-evolved with their hosts [13]. Indeed, these taxa lack environmental reservoirs. Other members of the phylum, such as *Flexibacter*, *Flavobacterium*, *Cytophaga*, and their relatives are associated with marine, soil, or other environmental habitats. There are exceptions: *Cytophaga* for instance has been detected in the gut microbiomes of non-Westerners [14], but whether they are passing through with food or are residents remains to be ascertained.

Each of these three genera of Bacteroidetes most commonly encountered in the Western gut microbiome (*Prevotella*, *Bacteroides*, *Porphyromonas*) are quite diverse. Currently 99 “species” of *Bacteroides* have been described in culture and their names accepted by the nomenclature. (Note that there is no definition of “species” for bacteria: Species names are inherited from cultured strains without a systematic definition; 97% pair-wise identity between 16S rRNA gene sequences is used as a species level-designation but this is somewhat arbitrary.) When a database such as Greengenes is explored, it is apparent that the 99 cultured *Bacteroides* species have a vast number of relatives that are known from their 16S rRNA gene sequences alone, and these flesh out the diversity of the *Bacteroides* clade. *Prevotella* is the most diverse of the three (in terms of total branch length in the Greengenes tree), however, it has far fewer (*n* = 51) described species compared to the *Bacteroides*. Since the properties of these uncultured organisms can only be inferred from their cultured relatives and their context, a great deal of their biology remains to be better characterized.

Within a given species (e.g., *Prevotella copri*), genomic diversity is high (“strains” refer to variants within species). For instance, metagenomic analyses of human stool samples have revealed very high levels of functional gene diversity in *P. copri* genomes recovered from the microbiomes of different individuals [15, 16]. These genomic differences are likely driven by pressure for niche differentiation in the face of competition in a crowded ecosystem. Indeed, within a body habitat, single-gene differences between genomes are sufficient to predict a strain’s carbohydrate preference [17]. Strains of *B. thetaiotaomicron* that differ in genome content have been shown to vary in their responses to diet in mice [18]. Members of the Bacteroidetes appear to have diversified their genome gene contents, resulting in a bewildering genomic diversity that matches their prowess in diverse carbohydrate utilization across habitats [13].

Despite their diversity at every level of resolution, members of the phylum Bacteroidetes share certain attributes that reflect their shared ancestry. Their genome content indicates a superlative ability to utilize polysaccharides [13]. Indeed, compared to bacteria of other phyla, Bacteroidetes members encode a proportionally high number of carbohydrate-active enzymes (CAZYmes such as glycoside hydrolases and polysaccharide lyases) that enable use of both dietary and host mucosal glycans [19]. They also frequently encode a signal peptide for enzyme export to degrade glycans that cannot penetrate the bacterial cell wall. For example, of *B. thetaiotaomicron*’s 280 glycan-cleaving enzymes, 230 have signal peptides, a higher proportion than other gut species [19]. So far, the greatest number of glycan degrading enzymes has been attributed to *B. cellulosilyticus*, totaling an astonishing 510 CAZYmes [18]. Among *Prevotella* species, 34 to 107 CAZYmes have been identified per genome [20]. The ability of these bacteria to take on a kaleidoscope of glycans makes them ideally suited to an omnivorous host with a variable diet.

It also allows them to be simultaneously generalists and specialists as they switch back and forth between substrate types. Members of the Bacteroidetes appear to be metabolically highly flexible. A recent proteomics analysis of obese and lean human gut microbiomes has highlighted that Bacteroidetes are more metabolically active in obese microbiomes despite their lower abundances [21]. Metabolic flexibility was demonstrated in a mouse model system by Sonnenburg and colleagues, who showed that when *B. thetaiotaomicron* was mono-associated with germfree mice, it exhibited a clear preference for certain substrates over others, and would alter gene expression to match changing substrate availability in its environment [22]. When its environment was depleted in polysaccharides from host food consumption, *B. thetaiotaomicron* switched its gene expression patterns towards enzymes capable of metabolizing host-derived mucus glycans [22]. When other bacterial species were introduced to the gut, *B. thetaiotaomicron* again adjusted its gene expression patterns [23].

There is a limit to the metabolic versatility of the *Bacteroides*, and indeed in some extreme conditions they start to fall in abundance. For instance, germfree mice colonized with human microbiota and fed a low-fat, plant polysaccharide-rich diet show a significant decrease in Bacteroidetes abundance upon a dietary shift to a high-fat, high-sugar “Western” diet [24]. But under physiologically normal conditions, these bacteria dynamically adjust their behavior in an ever-changing environment shaped by the host and by other members of the microbiome.

### *Bacteroides* vs. *Prevotella*

*Bacteroides*, *Prevotella*, and *Porphyromonas* are found throughout the human intestine. A majority of cultured *Prevotella* and *Porphyromonas* species are found in the mouth, and a few in the gut, whereas a majority of cultured *Bacteroides* species are found in the gut. Within the distal gut, species of *Bacteroides* and *Prevotella* may be antagonistic, and this is based on a few disparate observations. Sequence-based studies of gut bacterial diversity across human populations have shown that the relative abundance of the *Bacteroides* genus forms a gradient from low to high; in Westerners, it averages between 40 and 60% [25]. But a small minority of individuals harbors higher levels of *Prevotella* than *Bacteroides* [25, 26]. *Prevotella* is more common in non-Westerners who consume a plant-rich diet—as was recently shown for Papua New Guineans [27]—and has been linked to vegetarianism in Westerners [28]. The apparent trade-off between *Bacteroides* and *Prevotella* has formed the most compelling basis for the “enterotype” concept, wherein subjects are categorized into either the *Bacteroides* or *Prevotella* enterotypes based on which one is dominant [26]. This is a controversial approach to complexity (i.e., making a gradient into a binary system [25]); however it does highlight the very intriguing relationship between these two taxa in the gut. Kovatcheva-Datchary and colleagues investigated this relationship directly by competing *B. thetaiotaomicron* against *P. copri* in germfree mice—results of their work suggest an antagonism or competition, the basis of which is unclear [29].

## Bacteroidetes and diet

Diet is a major driver of microbiome diversity. Recent genetic studies have highlighted the importance of host genotype in determining the relative abundances of specific taxa in the gut microbiome [30–34], yet remarkably few of the Bacteroidetes are influenced by host genetics (i.e., are heritable). This implies that for the majority of the members of this phylum, environmental factors (which include diet) determine their relative abundances across hosts [31, 33]. Genetic studies in humans and mice have also highlighted the importance of environmental factors on Bacteroidetes abundances, since genetic factors are typically not important in explaining variance in their abundances across individuals in a population [31, 35]. This could imply that Bacteroidetes levels are so important to host health that the genes important for maintaining them went to fixation in mammalian evolution, leaving environmental influence to fine-tune their abundances within hosts. The strong influence of environmental factors on the patterns of distribution of Bacteroidetes across subjects implies that they are good targets for therapeutic interventions as their abundances may be tunable.

### Diet composition

Short-term diet studies have reported associations between Bacteroidetes relative abundance in stool and diets rich in animal foods (high fat, high protein) and low in fiber. High fecal *Bacteroides* abundance was linked positively with a diet rich in protein and animal fat and negatively with fiber intake in a 10-day study [28]. In a test of the effects of extreme (all animal products, all plant products) diets in ten volunteers, the animal-based diet rapidly drove an enrichment of *Bacteroides* and *Alistipes* species [36]. In contrast, levels of *Bacteroides* correlated positively with long-term patterns of fiber intake, not fat, in Finnish monozygotic twins with similar calorie intakes [37]. It is possible that short-term and long-term dietary studies highlight different aspects of the biology of these species. Their resilience in a high fat/protein diet may be related to their bile resistance [36], whereas long-term high fiber in the diet fosters a stable dominance. Reconciling the long-term and population-level patterns (i.e., Bacteroidetes associate with high fiber) with the short-term observations (i.e., they associate with protein intake) will require a better understanding of how these diets impact the digestive milieu (i.e., changes in bile, pH, substrate availability). However, the contradictory findings of studies asking similar questions may be also due to the inter-individual differences in microbiome composition.

### Overnutrition

Overnutrition refers to excess calorie intake over energy needed to maintain body weight, and there are suggestions that Bacteroidetes abundances are sensitive to this condition. Jumpertz and colleagues conducted an in-patient study of obese and lean individuals randomly assigned to diets of weight maintenance or overnutrition (2400 and 3400 Kcal/day, respectively). Overfeeding in lean subjects led to a 20% decrease in Bacteroidetes in stool concurrently with an increased energy harvest of roughly 150 Kcal [38]. A similar observation has been made in Finnish monozygotic twins, where excess energy intake was associated with reduced numbers of *Bacteroides* [37]. Interestingly, Roux-en-Y gastric bypass surgery resulted in an increase in *Bacteroides*, which may be attributed to reduced calorie load rather than weight loss [39]. These observations suggest that nutrient status impacts the Bacteroidetes with consequences for energy harvest efficiency. Early observations of low Bacteroidetes in obese mice and increasing levels when obese human subjects were dieting are consistent with Bacteroidetes responding to energy load [5–7].

### Undernutrition and fasting

Changes to the human gut microbiome under fasted conditions have yet to be explored, but microbial profiling under malnourishment has shown varied effects on Bacteroidetes abundance. In a study of Bangladeshi children, malnourishment was associated with depletion of Bacteroidetes [40]. However, not all members of the phylum followed this pattern; for instance, *Prevotella* was more abundant in healthy children and *Parabacteroides* in the malnourished [40]. A separate study of the same population revealed a reduction in *B. fragilis*, *B. galacturonicus*, and *P. copri* in nine malnourished Bangladeshi children compared to healthy children of a similar age [41]. It has been suggested that the lack of Bacteroidetes may contribute to malnourishment via a reduction in ability to ferment glycans and generate SCFAs [40]. A comparison of gut microbiomes of 13 Malawian twin pairs discordant for kwashiorkor (protein-dependent malnutrition) did not detect consistent patterns for Bacteroidetes members [42], although specific members may well contribute to disease.

Studies of fasting in mice have offered insights into changes in Bacteroidetes abundance during nutrient deprivation under controlled conditions. Mice fasted for 1 to 3 days have shown a significantly greater proportional representation of Bacteroidetes compared mice fed ad libitum [43, 44]. However, specific taxa can respond to changes in feeding frequency differently. For instance, rats restricted to a 1-hour feeding period for 6 days exhibited a significant increase in the relative abundance of *Bacteroides* and *Prevotella*, yet a significant decrease in the relative abundance of the Bacteroidetes phylum overall [45]. These nuances again reflect different dynamics for different phylum members.

### Feeding patterns

Diet intake is a strongly diurnal process and initial studies into this area indicate the possibility of circadian rhythms in microbiomes. In one such study, Zarrinpar et al. determined the diurnal effect on the murine gut microbiome by sacrificing mice every 4 h. Bacteroidetes levels peaked during the day when mice were fasting and dropped during nocturnal feeding [46]. However, this pattern does not appear consistent between studies. Thaiss et al. restricted mice with no functional host clock to a 12-h daytime or nighttime feeding period for 2 weeks, during which fecal microbiota samples were collected every 6 h for two light-dark cycles. *Bacteroides* abundances peaked within the 12-h feeding period, regardless of whether this feeding period was during the day or night [47]. In humans, the lowest daily *Parabacteroides* abundance occurred around midnight [47]. How these diurnal patterns are related to diet consumption patterns (e.g., amounts consumed at each feeding) remains to be understood.

## Bacteroidetes and metabolic disease

Among recent human studies of type II diabetic cohorts, metagenomic studies (in which bulk microbiome DNA is sequenced) have noted associations between certain species of *Bacteroides* and diabetes, although the patterns differ by study [48, 49]. In a study of 345 Chinese individuals, Qin et al. observed members of the genera *Bacteroides*, *Alistipes*, and *Parabacteroides* to be more abundant in type II diabetic subjects compared to controls with normal glucose metabolism [48]. A similar analysis of 53 age-matched Swedish women with type II diabetes and their controls noted enrichment of some *Bacteroides* species but depletion in others [49]. Women with high HbA1c, an indicator of poor blood glucose control, showed a decrease in abundance of *Bacteroides* species [49]. Together, these studies highlight the complexity of interactions, where diet, population genetics, health status, etc., may affect patterns of microbial ecology in the gut. Given the vast swath of diversity within this phylum, it is increasingly clear that not all members can be expected to interact with their host in the same ways.

Delving deeper into the relationships between individual taxa, diet, and glucose tolerance has further revealed species-specific effects. Zeevi et al. used implanted glucose sensors to continuously trace blood glucose response to macronutrient intake over a week in 800 participants [50]. The relative abundance of Bacteroidetes in stool was associated with a poor postprandial glucose response, though within the phylum, many species correlated with a positive postprandial glucose response. A subset of the cohort was fed personalized diets based on a multivariate model determined to contribute to reduced postprandial glucose response. On these diets, Bacteroidetes levels were health-associated and levels of *Bacteroides* species increased when individuals consumed the diet optimized to their microbiota, blood parameters, dietary habits, anthropometrics, and physical activity [50]. This study highlighted the importance of dietary context on the association of Bacteroidetes and health status.

Gnotobiotic animals (i.e., germfree animals inoculated with known microbial species) are ideally suited to testing the effects of particular species on host metabolism, as they control maximally for environmental conditions. Kovatecheva-Datchary et al. inoculated mice with *P. copri* and *B. thetaiotaomicron* singly and together [29]. Mice mono-associated with *P. copri* were more glucose tolerant than mice mono-associated with *B. thetaiotaomicron*. The *P. copri* associated mice exhibited greater levels of gene expression for the glycogen synthesis enzyme glucose-6-phosphatase (G6pc), suggesting that *P. copri* was able to affect host glucose metabolism by promoting hepatic glycogen storage. *B. thetaiotaomicron* mono-association led to the opposite phenotype: decreased hepatic glycogen storage, which was correlated with increased expression of the glycogen catabolic enzyme and glycogen phosphorylase. The mechanisms underlying the effects of these two gut bacterial species on glucose metabolism are unclear. However, this study provides a clear example of how two related species can have very different effects on host metabolism.

Another approach to testing the effects of single species on host metabolism in physiologically normal mice is daily dosing (akin to taking a probiotic). Dosing of conventionally raised mice with cultures of *B. uniformis* [51], *B. acidifaciens* [52], or *P. copri* [29] resulted in improved glucose tolerance and insulin sensitivity compared to dosing with heat-killed cultures as controls. Authors of these studies speculate that microbial metabolites could be effectors of improved metabolism. However, these potential effector molecules, and their modes of action, have yet to be identified.

## Conclusions

Since the initial observations of low Bacteroidetes levels in obesity, far more is known about the bacteria themselves, their distribution across hosts, and their response to diet and to their environments. The Bacteroidetes is a vast phylum with diversity at every level of resolution, from the so-called genus down to the genomes of strains. Members of the Bacteroidetes are highly adapted to life in a rapidly changing environment. Given their diversity within and across populations, it is not too surprising that phylum-level patterns in relation to a complex disease like obesity are not ubiquitous. For the most part, mechanisms by which specific members of the microbiota can affect human phenotypes remain to be elucidated. Great strides have been made to characterize species and strain specific effects on eliciting host responses, particularly in mouse models. One challenge to this effort is that a species may provoke an obesogenic response while still producing an obesity protective metabolite. It is likely that the presence of a microbe in the gut can result in multiple and sometimes opposing effects on host health. Resolution of the obesogenic potential of a species or strain belonging to the Bacteroidetes phylum, and the circumstances under which that potential is expressed, will be key into moving microbial profiling into the clinic. Moreover, greater genomic detail provided by metagenomic assemblies will allow for resolution of the varying functional capacities of strains inhabiting individual patients.

The prospect of health-interpretable microbiome data is an exciting one. Microbiome-based therapeutics must take into consideration the nuanced characteristics of a phylum like the Bacteroidetes if they are to be successful. Although much is yet to be uncovered about how an individual may interact with their gut microbiome to achieve beneficial health outcomes, a patient’s ability to decrease adiposity will be dynamically related to responses of their gut microbiota.
